# Diversity of cancer-related identities in long-term prostate cancer survivors after radical prostatectomy

**DOI:** 10.1186/s12885-021-08776-7

**Published:** 2021-09-20

**Authors:** Matthias Jahnen, Eike Mynzak, Valentin H. Meissner, Stefan Schiele, Helga Schulwitz, Donna P. Ankerst, Jürgen E. Gschwend, Kathleen Herkommer, Andreas Dinkel

**Affiliations:** 1 Department of Urology, Klinikum rechts der Isar, School of Medicine, Technical University of Munich, Ismaninger Str. 22, 81675 Munich, Germany; 2 Department of Mathematics, Technical University of Munich, Boltzmannstr. 3, 85748 Garching, Germany; 3 Department of Psychosomatic Medicine and Psychotherapy, Klinikum rechts der Isar, School of Medicine, Technical University of Munich, Langerstr.3, 81675 Munich, Germany

**Keywords:** Prostate cancer, Cancer-related identity, Cancer survivors, Survivorship, Psychosocial adaptation

## Abstract

**Background:**

Individuals affected by cancer need to integrate this experience into their personal biography as their life continues after primary therapy, leading to substantial changes in self-perception. This study identified factors uniquely associated with 5 different cancer-related identities in order to improve the understanding of how self-perception in men affected by prostate cancer is associated with certain clinical and psychosocial characteristics.

**Methods:**

In this cross-sectional study, long-term prostate cancer survivors after radical prostatectomy were asked to choose one of 5 cancer-related identities that described them best. Associations with sociodemographic, clinical, and psychological variables were investigated using multivariable logistic regression.

**Results:**

Three thousand three hundred forty-seven men (mean age 78.1 years) surveyed on average 15.6 years after prostatectomy were included. Most men favored the terms “someone who has had cancer” (43.9%) which was associated with a mild disease course, and “patient” (26.3%) which was associated with ongoing therapy and biochemical disease recurrence. The self-descriptions “cancer survivor” (16.8%), “cancer conqueror” (10.9%) and “victim” (2.1%) were less common. “Cancer survivor” was associated with high perceived disease severity (OR: 1.86 [1.44–2.40]). “Cancer survivor” and “cancer conqueror” were related to high benefit finding (OR: 1.89 [1.48–2.40], OR: 1.46 [1.12–1.89] respectively), and only “cancer conqueror” was associated with high well-being (OR: 1.84 [1.35–2.50]). Identification as “victim” was associated with a positive depression screening and low well-being (OR: 2.22 [1.15–4.31], OR: 0.38 [0.20–0.72] respectively) (all *p* < 0.05).

**Conclusions:**

Although long-term survival is common among men affected by PCa, they display a large diversity in cancer-related identities, which are associated with unique clinical and psychological characteristics. These cancer-related identities and their distinctive properties are associated with psychological well-being even after a long follow-up.

## Background

With an aging population along with improvements in early detection and treatment, the numbers of individuals diagnosed with cancer, as well as successfully treated, continue to increase [1]. In many cases cancer diagnosis and treatment become part of a continuing life story rather than its final chapter. However, living with cancer is in many cases still accompanied by a wide range of hardships deriving from factors such as pain, therapy side effects, accelerated aging, existential fears and distrust in one’s own body [2–5]. This has placed growing importance on advising individuals affected by cancer on how to process their cancer experience and how to integrate it into their personal biography [6, 7]. In this regard, particularly in the U.S., the concept of survivorship and identification as a “cancer survivor” has been advocated, while other characterizations, such as “patient” or “cancer victim”, which connotate with a passive stance, have become more and more outdated [6–8]. Accordingly, a shift away from these terms towards those that accentuate overcoming the disease, such as “cancer survivor” or “cancer conqueror”, have been proposed for medical practice [7].

Deimling et al. found that self-identification as “survivor” was associated with increased positive affect, benefit finding and well-being in various types of cancers and that when given the choice, individuals affected by cancer often preferred the term “cancer survivor” over “patient”. Consequently, they suggested that adapting a “survivor” identity is beneficial for mental health and disease coping [7]. However, subsequent research revealed that most individuals remain reluctant and identify more with the term “someone who has had cancer” than “cancer survivor” or “cancer conqueror” without a major drawback in overall well-being [8–10]. Therefore, it was suggested that actively trying to impose a cancer related identity (CRI) might cause more harm than benefit [11]. Additionally, it has been shown that individuals continuously affected by clinical or emotional symptoms after primary treatment for cancer tend to still perceive themselves as “patients” [12]. These mixed results complicate definitive recommendation of empowerment via specific CRIs.

Living with cancer is a major challenge for many men affected by prostate cancer (PCa). With 10-year survival almost unaltered in the majority of cases, long-term continuation of life after diagnosis has become the norm for men diagnosed with PCa [13, 14]. Nevertheless, regular follow-up visits, biochemical tumor recurrence rate of 20 to 50% 10 years after primary therapy, as well as side effects from primary and adjuvant therapy may be a burden for many of those men [15–17]. Thus, research on CRIs and their impact on well-being in men affected by PCa is of interest. Small US studies showed that 1 to 8 years after primary diagnosis, up to 35% of men affected by PCa favored the terms “cancer survivor” or “cancer conqueror “as self-description [8, 9]. While identification as a “survivor” was associated with positive affect, identification as a “victim” was associated with negative affect. Results suggested that lower threat appraisal, thoughtful reflection and gaining an understanding through peers might be contributing factors in adopting a “survivor” identity [7–9]. However, data on clinical factors as well as psychological characteristics that might accompany the development of certain CRIs, other than a “cancer survivor” one, are still lacking.

This study assessed how men affected by PCa self-identify with regards to 5 different CRIs: “patient”, “victim”, “someone who has had cancer”, “cancer survivor” and “cancer conqueror”. These 5 CRIs carry different connotations with regards to one’s personal cancer experience, which range from passive and submissive to actively engaging. Assessment of such a balanced set of CRIs allowed a detailed comparison between men, who adapt an active CRI, with men, who identify with a submissive or a neutral CRI. Furthermore, associations of these CRIs with a broad set of sociodemographic characteristics, clinical factors, and psychosocial aspects were investigated, using data from a large German PCa database.

## Methods

### Design and procedure

Data were gathered as part of the German research project ‘Familial Prostate Cancer’, which since 1993 has prospectively recruited PCa patients regardless of a family history of PCa via collaborating urologists and clinics. Further details about the research project and its multi-centric database has been described elsewhere [15, 18]. In short, participants receive annual questionnaires (conducted in German) concerning clinical, sociodemographic, and psychosocial information, with further clinical information being obtained through the corresponding treating urologist. The ethic committee of the TU Munich has approved this research project.

For the present study, cross-sectional data from the annual follow-up of 2019 were analyzed. By January of 2020, 4141 of 6168 participants (67.1.0%) had returned the questionnaire. From these, only participants who underwent radical prostatectomy as primary treatment and who answered the item regarding cancer identity with a single answer were included (*N* = 3347).

### Measures

#### Cancer-related identity

Following previous research on men affected by PCa, participants were asked to choose only one of the following 5 CRIs describing them most suitable with regards to their cancer experience [7, 8, 11]: “patient”, “victim”, “someone who has had cancer”, “cancer survivor” and “cancer conqueror”.

#### Sociodemographic and clinical characteristics

Sociodemographic data included: age at survey, school education, current partnership, and children. Clinical data included age at surgery, time since surgery, presence of a second primary cancer, family history of PCa (yes: at least one consanguine relative with PCa vs. no), PSA level at diagnosis, histopathological Gleason-Score, histopathological grading, organ-confined stage at RP according to TNM classification of 2002, biochemical recurrence (PSA level ≥ 0.2 ng/ml) during follow-up, biochemical recurrence at survey, discontinued PSA follow-up and ongoing PCa treatment at survey.

#### Depression and anxiety

Symptoms of depression and anxiety were assessed using the validated ultra-brief instruments Patient Health Questionnaire-2 (PHQ-2) and General Anxiety Disorder-2 (GAD-2) scale. For both scales (range 0–6), a cut-off score ≥ 3 indicates a clinical level of depression or anxiety, respectively [19, 20]. Cronbach’s alpha coefficients for PHQ-2 and GAD-2 scale were 0.65 and 0.75 respectively, representing satisfactory internal consistency.

#### Perceived severity of the disease

The perceived severity of being affected by PCa was assessed with the single item “Having had prostate cancer is one of the worst things that happened to me in my life” (adapted from [21] using past tense). Participants were asked to answer on a four-point scale ranging from `strongly disagree´ (1) to ‘strongly agree’ (4). Responses (1) and (2) and responses (3) and (4) were combined to ´low perceived severity´ and `high perceived severity´, respectively.

#### Benefit finding

Benefit finding was assessed using one item with high factor loading and high face validity adapted from the German version of the 17-item benefit finding scale: “My prostate cancer has helped me become more focused on priorities, with a deeper sense of purpose in life” [22, 23]. Participants were asked to answer on a five-point scale ranging from ‘not at all’ (1) to ‘extremely’ (5). Responses (1) and (2) and (3) to (5) were combined to ´low benefit finding´ and `high benefit finding´, respectively [24].

#### Well-being

Well-being was assessed using the single item” How much of the time during the past month did you feel happy” [25]. Participants were asked to answer on a four-point scale ranging from ‘none’ (1) to ‘all’ (4). Responses (1) and (2) and (3) and (4) were combined to `low well-being´ and `high well-being´, respectively.

### Statistical analysis

Descriptive statistics were calculated for all study variables. Chi-square and Wilcoxon tests were applied for analyzing associations between the CRIs and sociodemographic, clinical, and psychological variables. Multivariable logistic regression with backward elimination was used to identify variables independently associated with each of the CRIs. All psychosocial and sociodemographic variables as well as certain clinical characteristics (time since surgery, family history of PCa, second primary tumor, biochemical tumor recurrence during follow-up and survey, ongoing therapy) were included in multivariable logistic regression analysis. Statistical significance was set at *p* < 0.05. All analyses were performed using SAS (Version 9.4).

## Results

**Fig. 1:**
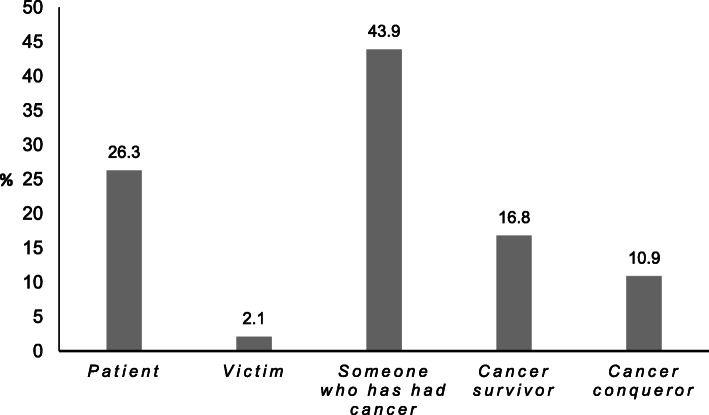
Self-identification in the study sample of men affected by prostate cancer with a mean follow-up of 15.6 years

Three thousand three hundred forty-seven men affected by PCa with a mean age at survey of 78.1 years (standard deviation (SD) = 6.3) and a mean follow-up of 15.6 years (SD = 3.8) were included in the analysis (Table 1). Men self-identified most frequently as “someone who has had cancer” (43.9%) followed by “patient” (26.3%). The terms “cancer survivor” and “cancer conqueror” were favored by 16.8 and 10.9%, respectively. “Victim” was the least endorsed term (2.1%) (Fig. 1).

**Table 1:** Sociodemographic, clinical and psychological characteristics of the study sample (*n* = 3347)

	n	%
Sociodemographic factors		
*Age at survey (years)* M = 78.1, SD = 6.3		
≤ 70	375	11.2
> 70 ≤ 80	1599	47.8
> 80	1373	41.0
*Educational level*		
primary, secondary - low	1187	39.8
secondary - intermediate	519	17.4
secondary - high	362	12.2
tertiary	911	30.6
*Partnership*		
yes	3053	92.1
no	260	7.9
*Children*		
0	376	11.5
≥ 1	2901	88.5
Clinical characteristics		
*Age at* surgery (years) M = 62.5, SD = 6.1		
≤ 55	399	11.9
> 55 ≤ 65	1742	52.1
> 65	1206	36.0
*Time since surgery (years)* M = 15.6, SD = 3.8		
≤ 10	198	5.9
> 10 ≤ 15	1425	42.6
> 15 ≤ 20	1349	40.3
> 20	375	11.2
*Second primary cancer*		
yes	430	12.9
no	2917	87.1
*Family history of PCa*		
yes	1297	38.8
no	2050	61.2
*PSA level at diagnosis (ng/ml)*		
≤ 4	307	9.9
> 4 ≤ 10	1870	60.1
> 10	934	30.0
*Gleason score*		
2–6	1424	50.5
7, 3 + 4 = 7, 4 + 3 = 7	1131	40.0
8–10	269	9.5
*Grading*		
G I	133	4.1
G II	2292	70.3
G III	833	25.6
*Organ-confined stage at RP*		
yes	2343	71.0
no	955	29.0
*Biochemical recurrence during follow-up*		
yes	1211	40.9
no	1749	59.1
*Biochemical recurrence at survey*		
yes	572	20.0
no	2295	80.0
*Discontinued PSA follow-up*		
yes	186	5.6
no	3161	94.4
*Ongoing treatment at survey*		
yes	423	12.9
no	2864	87.1
Psychosocial factors		
*PHQ-2 (depression screening)*		
positive screening (≥3)	469	14.8
negative screening (< 3)	2707	85.2
*GAD-2 (anxiety disorder screening)*		
positive screening (≥3)	360	11.4
negative screening (< 3)	2798	88.6
*Perceived severity of disease*		
*low*	1448	45.0
*high*	1770	55.0
*Well-being*		
*low*	1075	34.2
*high*	2067	65.8
*Benefit Finding*		
*low*	1823	56.0
*high*	1429	44.0

Note: *M* mean, *SD* standard deviation, *PCa* prostate cancer, *PSA* prostate specific antigen, *RP* radical prostatectomy, *PHQ* patient health questionnaire, *GAD* general anxiety disorder

Men who self-identified as “someone who has had cancer” were the youngest at survey, while men who self-identified as “cancer survivor” were the oldest. Other sociodemographic variables such as partnership or children did not show any significant differences between the different CRIs. Men who self-identified as “someone who has had cancer” had the lowest percentage of biochemical recurrence during follow-up (31.6% vs. 40.9% on average). High perceived severity of the disease was reported most frequently by men self-identified as “cancer survivor” (68.9%) and “victim” (82.2%). While men self-identified as “cancer conqueror” expressed most often high well-being (75.2%), men self-identified as “cancer survivor” and “victim” expressed high well-being least often (59.0 and 34.8%, respectively). High benefit finding was found most often in men self-identified as “cancer survivor” or “cancer conqueror” (56.1, 51.4%, respectively vs 44.0% on average). (all *p* < 0.0001) (Table 2).

**Table 2:** Comparison of key characteristics of the 5 cancer-related identities

	*Someone who has had cancer n = 1468 (%)*	*Patient* *n = 882* *(%)*	*Cancer survivor n = 561* *(%)*	*Cancer conqueror n = 364* *(%)*	*Victim* *n = 72* *(%)*
Age at survey (years)**					
≤ 70	12.7	10.2	7.8	13.2	9.7
> 70 ≤ 80	49.4	48.2	43.5	45.9	52.8
> 80	37.9	41.6	48.7	40.9	37.5
Educational level**					
primary, secondary - low	36.3	38.8	46.0	45.1	52.5
secondary - intermediate	17.9	17.3	15.4	19.0	16.4
secondary - high	12.5	11.9	12.8	10.2	13.1
tertiary	33.3	32.0	25.8	25.7	18.0
*Partnership*					
yes	92.2	91.9	90.7	95.0	91.4
no	7.8	8.1	9.3	5.0	8.6
*Children*					
0	12.1	12.8	9.3	9.9	8.5
≥ 1	87.9	87.2	90.7	90.1	91.5
*Age at* surgery *(years)*					
≤ 55	13.8	10.0	8.9	13.5	12.5
> 55 ≤ 65	52.1	51.7	51.7	52.7	55.6
> 65	34.1	38.3	39.4	33.8	31.9
Time since surgery (years)***					
≤ 10	5.7	6.8	5.5	5.8	4.2
> 10 ≤ 15	44.3	42.2	36.0	46.4	43.0
> 15 ≤ 20	40.7	40.8	41.7	34.9	41.7
> 20	9.3	10.2	16.8	12.9	11.1
*Second primary cancer*					
yes	11.7	12.5	15.5	13.7	16.7
no	88.3	87.5	84.5	86.3	83.3
*Family history of PCa*					
yes	40.5	35.9	38.2	40.4	33.3
no	59.5	64.1	61.8	59.6	66.7
Biochemical recurrence during FU****					
yes	31.6	49.1	51.3	39.6	54.1
no	68.4	50.9	48.7	60.4	45.9
Biochemical recurrence at survey****					
yes	13.7	29.6	21.9	16.8	25.9
no	86.3	70.4	78.1	83.2	74.1
*Discontinued PSA follow-up*					
yes	5.3	5.1	6.6	5.5	9.7
no	94.7	94.9	93.4	94.5	90.3
Ongoing treatment at survey****					
yes	6.2	19.7	18.3	13.0	23.2
no	93.8	80.3	81.7	87.0	76.8
PHQ-2 (depression screening) ****					
positive screening (≥3)	12.0	15.0	19.7	13.0	39.1
negative screening (< 3)	88.0	85.0	80.3	87.0	60.9
GAD-2 (anxiety disorder screening) ****					
positive screening (≥3)	9.7	10.2	16.2	8.7	37.3
negative screening (< 3)	90.3	89.8	83.8	91.3	62.7
Perceived severity of disease****					
low	48.4	50.6	31.1	44.5	18.8
high	51.6	49.4	68.9	55.5	82.2
Well-being****					
low	32.3	34.5	41.0	24.8	65.2
high	67.7	65.5	59.0	75.2	34.8
Benefit finding****					
low	59.6	60.6	43.9	48.6	58.6
high	30.4	39.4	56.1	51.4	41.4

Note: **** *p* < .0001; *** *p* < .001; ** *p* < .01; * *p* < .05; *PCa* prostate cancer, *FU* follow-up, *PSA* prostate specific antigen, *PHQ* patient health questionnaire, *GAD* general anxiety disorder, *per.* perceived

Multivariable logistic regression analyses showed almost no associations between sociodemographic factors and the 5 CRIs. The two exceptions were an association between a “survivor” identity and higher age at survey and an association between identification as “someone who had cancer” and higher school education. Although associations between clinical factors and the CRIs were seldom, there were the following notable exceptions. While men identifying as “someone who has had cancer” were less likely to have faced a more complicated follow-up (biochemical recurrence or ongoing therapy), men identifying as “patient” were more likely to experience a current disease progress or ongoing therapy. Men with a “cancer survivor” identity were more likely to have experienced a biochemical recurrence during follow-up, but were less likely to have a current biochemical recurrence at survey (Table 3) (all *p* < .05).

**Table 3:** Factors associated with cancer-related identities in multiple logistic regression analysis

	*Someone who has had cancer* *OR [95% CI]*	*Patient* *OR [95% CI]*	*Cancer survivor* *OR [95% CI]*	*Cancer conqueror* *OR [95% CI]*	*Victim* *OR [95% CI]*
*Age at survey (years) [ref: ≤70]*					
> 70 ≤ 80	–	–	1.47 [0.98–2.22]	–	–
> 80	–	–	2.11 [1.39–3.20]	–	–
*Educational level [ref: primary, sec. low]*		–			
Secondary intermediate	1.37 [1.07–1.76]	–	–	–	–
Secondary high	1.16 [0.87–1.54]	–	–	–	–
Tertiary	1.34 [1.09–1.65]	–	–	–	–
*Family history of PCa [ref: no]*					
yes	1.20 [1.00–1.43]	–	–	–	–
*Biochemical recurrence during FU [ref: no]*					
yes	0.56 [0.46–0.68]	–	1.97 [1.49–2.61]	–	–
*Biochemical recurrence at survey [ref: no]*					
yes	–	1.93 [1.53–2.44]	0.68 [0.48–0.95]	–	–
*Ongoing treatment at survey [ref: no]*					
yes	0.37 [0.27–0.51]	1.90 [1.45–2.47]	–	–	2.07 [1.06–4.04]
*PHQ-2 (depression screening) [ref: negative screening (< 3)]*					
positive screening (≥3)	–	–	–	–	2.22 [1.15–4.31]
*Perceived severity of disease [ref: low]*					
high	–	0.75 [0.61–0.91]	1.86 [1.44–2.40]	–	–
*Well-being [ref: low]*					
high	–	–	0.77 [0.61–0.99]	1.84 [1.35–2.50]	0.38 [0.20–0.72]
*Benefit finding [ref: low]*					
high	0.79 [0.66–0.94]	0.75 [0.62–0.92]	1.89 [1.48–2.40]	1.46 [1.12–1.89]	–

Note: *PCa* prostate cancer, *PHQ* Patient Health Questionnaire, *FU* follow-up, *ref* reference, *OR* odds ratio, *CI* confidence interval, *sec.* secondary

Associations with psychosocial factors were present for all CRIs. Whereas men with a “cancer survivor” or “cancer conqueror” identity were more likely to be considered to have high benefit finding, men identifying as “someone who has had cancer” or “patient” were less likely to be considered to have high benefit finding. Self-identification as “cancer survivor” was also associated with an increased likelihood of high perceived disease severity and a decreased likelihood of high well-being. “Cancer conqueror” was the only CRI associated with an increased likelihood of high well-being. Men self-identified as “victim” were more likely to report high perceived disease severity and have a positive screening for depression. The identities “conqueror” and “victim” were associated with the least number of significant factors (Table 3) (all *p* < .05).

## Discussion

During the past decades growing focus has been placed on assisting individuals diagnosed with cancer on coping with the disease as they continue their life beyond the stage of primary therapy and successful recovery. In this regard it has been shown that there are different ways of perceiving this experience, leading to a variety of cancer-related identities (CRIs). Especially meaning making by adopting an active CRI and identifying as a “cancer survivor” has been advocated as beneficial [7–9].

In this analysis of men affected by prostate cancer (PCa) with a long follow-up after radical prostatectomy, most men self-identified with the more neutral terms “someone who has had cancer” or “patient”, while only one forth self-identified as “cancer survivor” or “cancer conqueror”. Only few men believed that “victim” would describe them best. These results are in accordance with previous research on men affected by PCa showing that, while the majority of such men favors a more neutral term such as “someone who has had cancer” as self-description, identification with regards to a cancer experience may vary widely [8, 9]. Nevertheless, it has to be pointed out that the endorsement of a “cancer survivor” identity was relatively low in our sample compared to previous research, which has mostly been done in the US [8, 11]. Outside of the US the term “cancer survivor” has been advocated less publicly as a positive empowering term. It has been shown that without this cultural background individuals affected by cancer might associate the term “survivor” or its literal translation with survival of natural disasters or military conflict and deem it as inappropriate to their situation [26–28]. This might have led to the low endorsement of the term in our sample of German men affected by PCa. However, as we applied a forced choice response, we do not know whether men would find other self-descriptions nearly equally appropriate. Thus, CRI not chosen do not necessarily imply negative appraisals.

Results indicated that men who preferred the more neutral term “someone who has had cancer” were more likely to have experienced PCa with an oncological uneventful follow-up (no biochemical recurrence) and less likely to report profound, positive changes derived from their cancer experience (benefit finding). These findings support previous studies finding that individuals who preferred a more neutral term considered their disease often as something unthreatening of their past and “hardly ever think about their disease” [10]. For men who self-identified as “someone who has had cancer”, PCa did not hold the centrality in their life sufficient to trigger the development of an active CRI [29]. Since PCa is generally linked with long-term survival, many men prefer “someone who has had cancer” over terms such as “cancer survivor” as self-description in the present as well as in previous studies [11].

A characteristic adversity of PCa is that, despite an excellent survival prognosis, biochemical cancer recurrence, requiring subsequent therapy, is seen in a considerable number of cases even 10 years after primary therapy [17, 30]. In this study sample at least a fifth reported a biochemical cancer recurrence at survey and/or an ongoing therapy. Men in this situation, characterized by cancer as a current medical condition rather than a previous life event, were more likely to perceive themselves as “patient”. This agrees with a study from Thong et al., showing that treatment, cancer recurrence or lingering cancer/therapy-related symptoms after primary cancer therapy were associated with self-identification as “patient” [12]. Men who self-identified as “patient” were less likely to consider their disease to have high severity, and no association between self-identification as “patient” and psychological distress was found. In contrast to previous studies, this suggests that identification with the more passive term “patient” is not necessarily associated with a more demanding disease burden and that a general disapproval of the term for individuals affected by cancer might be premature [10, 12].

In scientific literature individuals affected by cancer following primary therapy are regularly referred to as “cancer survivors” [27, 28]. The term is often used for certain time frames (most often 3 or 5 years) without signs of cancer recurrence, which indicate that individuals affected by cancer might be considered as cured. With growing research on cancer survivorship, the term “cancer survivor” has also been advocated as proper description of individuals affected by cancer regardless of their disease course in order to emphasize resilience and personal strength in one’s “fight” against cancer [28, 31]. Especially in the U.S., such an interpretation of the term “cancer survivor” has been promoted and individuals affected by cancer have been encouraged to adopt the term as self-description [11]. Some studies on cancer survivorship and CRIs have already indicated that adapting the identity of a “survivor” is a sign of actively engaging and coping with the cancer experience, which might lead to psychological benefits for the affected individual [7].

Analyses of narrative data have suggested, on the one hand, that identification as “cancer survivor” is often based on having experienced the disease as a serious life event and, on the other hand, a feeling of having successfully overcome the disease [7, 32]. These aspects are supported by the findings of this study showing that men who self-identified as “cancer survivor” were inclined to report high perceived disease severity. Further, these men were also more likely to have endured a biochemical recurrence during follow-up, while simultaneously being more likely to be biochemical recurrence-free at survey. This supports that overcoming a subjectively and objectively more stressing disease course may lead to the endorsement of a “survivor” identity. Moreover, previous research has suggested that adapting a “survivor” identity may bring forward positive changes driven by the disease experience [7, 10]. These assumptions are supported by findings of this study showing an association between a “survivor” identification and high benefit finding. However, men favoring the self-description as “survivor” were also less likely to report high well-being. These results contradict previous findings proposing a positive effect of adopting a “survivor” identity on psychological health and well-being. Consequently, findings here show that the implications of a “survivor” orientation may vary between individuals affected by cancer.

A previous review of CRIs pointed out that acceptance of the term “cancer survivor” derives partly from positive portrayal of the concept by survivorship movements in the media and support groups [11]. It should be noted that cancer survivor culture and research on CRIs is mostly based in the U.S., while cultivation of the term “survivor” or its equivalent translations is rather seldom in Europe [11, 33]. Limited exposure to a positive depiction of survivorship might lead to interindividual and intercultural differences in the understanding of the term and concept [34]. One should consider that without reference some individuals may choose a “cancer survivor” identity not as a sign of active coping, but rather to reflect the burden of being confronted by fundamental changes and a sense of near defeat derived from their cancer experience. Therefore, identification as “cancer survivor” might be for some an expression or even a cause of reduced psychological well-being.

Since the term “cancer survivor” and its literal translation might be subject to interpretation depending on the cultural background, the term “cancer conqueror”, which puts further emphasis on actively engaging and defeating the disease, was also included as potential CRI in this analysis. Previous studies that have investigated the term “cancer conqueror” have shown that endorsement of such an energetic CRI might be associated with positive affect and psychological well-being [8, 11]. In contrast to identification as a “cancer survivor”, which seems to be influenced by an objectively and subjectively demanding disease course, endorsement of the term “cancer conqueror” was not associated with any clinical factors or the perceived disease severity. This suggests that adapting this kind of identity might not be based on a certain disease course but rather on individual traits influencing the general outlook on life. This is supported by findings here showing that identification as a “cancer conqueror” is associated with high well-being as well as high benefit finding, which may reflect a generally optimistic attitude.

As in previous studies of men affected by PCa only a minority self-identified with the term “victim”, stressing the seemingly pitiful fate of individuals diagnosed with cancer [8, 9]. Most studies on the subject have found that endorsement of this submissive CRI is associated with psychological distress [7–10]. This is supported by the findings from this analysis showing that men self-identifying as “victim” were more likely to perceive the severity of their disease as high, and were more likely to have positive depression screening and low well-being. Our statistical findings have to be treated with caution due to the low number of men identifying as “victim”, nevertheless these men seem to be burdened most by their cancer experience and seem in need of further psychological support, even years after primary diagnosis and therapy.

Study findings must be considered within the limitations of the analysis. Limited by the cross-sectional design, causal assumptions on development of CRIs should be further investigated in longitudinal studies. Further, self-perception with regard to one’s cancer experience might also be a dynamic process and changes over time should be expected. Such developments cannot be observed by the applied study design and require further investigation. Nevertheless, it should be noted that multi-variable logistic regression analysis for all 5 CRIs was controlled for the time since surgery, but did not yield any significant results with regards to this parameter. This suggests that in our sample of men with a rather long follow-up after primary therapy, CRIs may be considered as somewhat stable. By only including men primarily treated with radical prostatectomy generalization towards all men affected by PCa is limited and implications for other cancer types should be treated with caution. Further, it has to be noted that limited by the available space of the annual questionnaires some psychological variables were measured using single items. Although this might decrease the validity of our measured psychological variables, previous research has shown that using single items can produce highly credible measurements when longer questionnaire cannot be applied [35, 36]. The variety in CRIs suggests that different terms may be equally appropriate in addressing men affected by PCa and that, though these men select a preferred identity when prompted, the remaining identities do not necessarily have negative connotations, and when given the choice some men would have picked several identities to describe themselves with regard to their cancer experience. Nevertheless, the CRIs were distinctively associated with different clinical circumstances and psychological factors, implying a clinically relevant differences in CRIs of men affected by PCa.

## Conclusion

Although long-term survival is common among men affected by PCa, they display a large diversity in CRIs that show unique associations with mainly clinical and psychological characteristics. These CRIs and their distinctive properties are present and seem to interact with psychological well-being even after a long follow-up. This demonstrates that an individual approach, which should include the evaluation of the patient’s cancer related identity, is essential in the post-clinical care of men affected by PCa in order to understand the personal impact of their cancer experience.

## Data Availability

This manuscript contains all associated data.

## Abbreviations

CRI: Cancer-related identity
PCa: Prostate cancer
